# Non-interleaved chiral metasurfaces and neural networks enhance the spatial resolution of polarimetry

**DOI:** 10.1038/s41377-024-01397-2

**Published:** 2024-02-20

**Authors:** Jaewon Jang, Minsu Park, Yeonsang Park

**Affiliations:** 1 Department of Physics, Chungnam National University, Daejeon, 34134 Korea; 2 Institute of Quantum Systems, Chungnam National University, Daejeon, 34134 Korea

**Keywords:** Metamaterials, Imaging and sensing

## Abstract

Non-interleaved chiral metasurfaces for high-spatial-resolution polarimetry are proposed and demonstrated. Furthermore, a convolutional neural network is incorporated to analyze interferometric images with the polarization state of light, and it results in accurate Stokes parameters.

## Abstract (web summary)

Polarimetry using non-interleaved chiral metasurfaces shows high spatial resolution with the help of convolutional neural networks.

## Graphical abstract

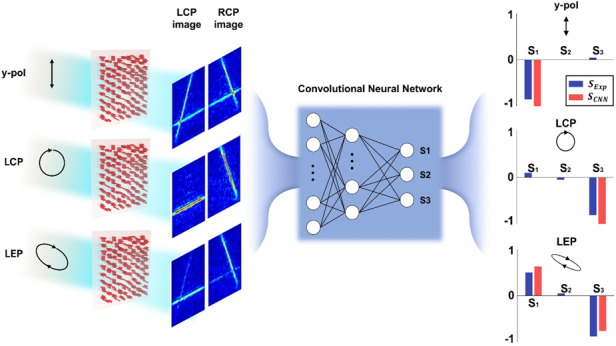

Analyzing the polarization of light provides a wealth of information beyond the structure and composition of materials. Currently, polarization analysis methods, including polarization spectroscopy, ellipsometry, and chiral sensing, are widely employed in numerous fields of photonics. However, achieving precise polarization measurements in many applications requires the integration of numerous optical components, inevitably leading to bulky measurement systems and increased costs^1,2^.

On the other hand, utilizing a polarimetry based on metasurfaces offers the potential to significantly reduce the size of polarization measurement systems^3–5^. Metasurfaces are arrays of nanostructures with subwavelength period that have the ability to modulate the amplitude, phase, and polarization of light^6–8^. Numerous groundbreaking metasurfaces have been demonstrated such as lenses^9–11^, beam generators^12,13^, quarter- and half-wave plates^14,15^,crcular polarization splitters^16,17^, holograms^18^ etc. Among these applications, metasurface-based polarimetry presents not only compact integration with other devices but also the possibility of versatile functions that conventional individual optical components can not achieve, sometimes even overcoming the inherent limitations of conventional optical elements^19^.

In previous studies on metasurface-based polarization measurement, various methods have been proposed. These include an in-line polarimeter using a single 2D array of rod antennas for the detection of polarization-selective directional scattering^20^, a technique that simultaneously measures diffraction contrasts of reflected light to determine the polarization state of incident light^3^, and polarization beam-splitting methods that characterize incident polarization states by measuring the intensities of refracted light spots^21^. In many cases, segmented-type metasurfaces^22^ or interleaved-type metasurfaces^23^ have been used to measure independent channels for light with different polarization states. These types of polarimetry are inevitable in spatial resolution decrease as the number of channels to be implemented increases.

In 2015, Arbabi et al.^24^ proposed metasurfaces that can completely control both phase and polarization by combining the dynamic phase and geometric phase, thus breaking the phase-spin coupling relationship. Similarly, Xu et al. demonstrated a polarization beam splitter by utilizing this spin-decoupled phase control method to split light at asymmetric angles^25^, and also showcased a vector beam generator^26^. On the other hand, Chen Chen et al. designed a spin-decoupled metasurface based on chiral meta-atoms and demonstrated spin-decoupled holographic imaging^27^. These chiral meta-atoms allow independent phase control without the need for structural rotation, as phase modulation relies solely on the geometric deformation of the structure. Therefore, independent phase control is achievable regardless of the orientation of the two spin lights. In a newly published paper in *Light: Science & Applications*, a polarimetry method based on this spin-decoupled chiral metasurface has been proposed and demonstrated^28^.

As illustrated in Fig. 1, Chen et al. designed a non-interleaved-type chiral metasurface where all meta-atoms can contribute to increase the efficiency of polarized light measurement, and demonstrated a method to measure the polarization state by simultaneously acquiring amplitude and phase information through interferometric techniques. Non-digitized measurement of polarization state of light using non-interleaved metasurfaces has advantages in efficiency because all incident light contributes to find out the Stokes parameters. Nevertheless, this method has difficulty in analyzing subtle and a little difference between triple interferometric intensities. By employing a convolutional neural network (CNN), they enhanced the capability of detecting the state of polarization (SoP) and achieved a high spatial resolution. The proposed polarimetry resultantly obtains information about the polarization state of incident light in the form of an image. Therefore, the CNN technique, which shows excellent performance in comparing and analyzing images, is very suitable for deriving Stokes parameters through non-digitized images. Recently, cutting-edge computing technologies such as artificial intelligence, machine learning, and deep learning have been accelerating the advancement of nanophotonics^29,30^. Now, the successful integration of two high-tech technologies, CNN and non-interleaved chiral metasurfaces, has made it possible to realize a very small-sized device capable of precisely measuring the input polarization state with high spatial resolution. In the future, this is expected to provide a very small and lightweight platform for various advanced fields that should measure polarization state of light, and this work will open a new path for the emergence of several applications using the polarization information of light in daily life.

**Fig. 1:**
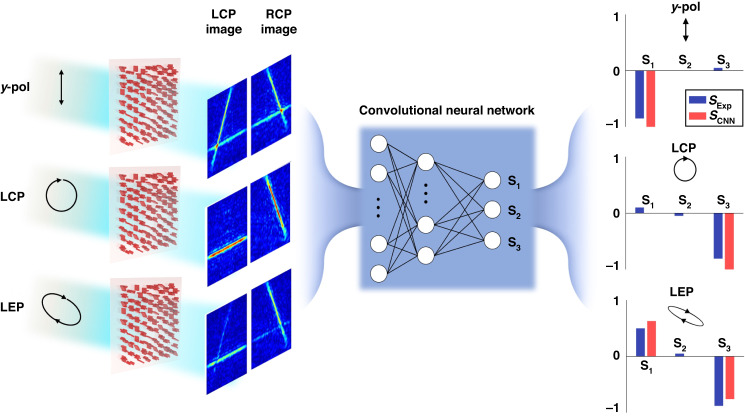
Schematics of the proposed non-interleaved chiral metasurface for a polarimetry with high spatial resolution assisted by a convolutional neural network (CNN) analysis. (Left) 3D schematics of the non-interleaved chiral metasurfaces and images measured when light of different polarization states is incident. y-pol: vertical polarization, LCP left-hand circular polarization, LEP left-hand elliptical polarization. (Middle) Schematic diagram of CNN. (Right) Comparison of Stokes parameters measured with the proposed polarimeter and analyzed with CNN. Measured images reproduced from ref. ^28^
